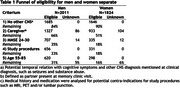# Women are less likely to be eligible for AD trials than men.

**DOI:** 10.1002/alz70859_104840

**Published:** 2025-12-25

**Authors:** Lieza G. Exalto, Siti S. Syaziyah, Xiaotian T Fang, Niels D. Prins, Sietske A.M Sikkes, Everard G.B. Vijverberg, Yvonne M.F. Lim, Wiesje M. van der Flier

**Affiliations:** ^1^ Julius Clinical, Zeist Netherlands; ^2^ Alzheimer Center Amsterdam, Neurology, Vrije Universiteit Amsterdam, Amsterdam UMC location VUmc, Amsterdam Netherlands; ^3^ Institute for Clinical Research, National Institutes of Health, Ministry of Health Malaysia, Shah Alam, n.a. Malaysia; ^4^ Brain Research Center, Amsterdam Netherlands

## Abstract

**Background:**

Fewer women participate in Alzheimer Disease (AD) trials than expected relative to their estimated representation in the global dementia population. Biotech, pharma, and patient organisations all aim for inclusive and representative trials, mainly focusing on participant engagement and recruitment. We aimed to explore the influence of trial design, by investigating the proportion of men and women presenting at a memory clinic, eligible for trial participation, based on commonly used inclusion criteria.

**Method:**

Clinical study registration was downloaded from clinicaltrials.gov (2023‐09‐08) to extract eligibility criteria of 563 phase II and III AD drug trials. A randomly selected 113 (20%) trials were manually annotated by three independent raters. Overall inter‐annotator agreement was >0.70 Cohen’s Kappa. Next, we applied the top 5 most common criteria in a stepped approach to women and men with a diagnosis of AD or MCI of Alzheimer Center Amsterdam.

**Result:**

Top 5 most common AD clinical trial eligibility criteria were 1) no other central nervous system (CNS) disorder related to cognition (84%), 2) participation of a caregiver (72%), 3) MMSE (66%; most common 24‐30), 4) no contra‐indications for study procedures (61%), 5) age (53%; most common 55‐85). In the period 2000‐2024 in total 3835 patients (48% women) with AD or MCI were seen for a first diagnostic visit at Alzheimer Center Amsterdam. After screening for 1) no other CNS disorder 84% of men and 90% of women remained eligible. Additional screening on 2) participation of caregiver reduces eligibility to 66% of men and 51% of women. 3) MMSE (24‐30) reduces eligibility to 35% of men and 18% of women. 4) no contra‐indications for study procedures reduces eligibility to 33% of men and 18% of women. Lastly, applying 5) age (55‐85) a final 31% of men and 16% of the women remain eligible (table 1).

**Conclusion:**

Based on commonly used inclusion criteria of clinical trials, women are less likely to be eligible for participation in AD drug trials than men. This discrepancy seems mainly related to lower MMSE at presentation and lack of caregiver presence. These results provide clues for design of trials to be alert for equal inclusion of women.